# The role of folate receptor alpha (FR*α*) in the response of malignant pleural mesothelioma to pemetrexed-containing chemotherapy

**DOI:** 10.1038/sj.bjc.6605501

**Published:** 2010-01-05

**Authors:** J E Nutt, A R A Razak, K O'Toole, F Black, A E Quinn, A H Calvert, E R Plummer, J Lunec

**Affiliations:** 1 Northern Institute for Cancer Research, Newcastle University, Framlington Place, Newcastle upon Tyne NE2 4HH, UK; 2 Department of Pathology, Royal Victoria Infirmary, Queen Victoria Road, Newcastle upon Tyne NE1 4LP, UK; 3 Northern Centre for Cancer Care, Freeman Hospital, Freeman Road, Newcastle upon Tyne NE7 7DN, UK

**Keywords:** Folate receptor *α*, mesothelioma, pemetrexed, cell lines, immunohistochemistry

## Abstract

**Background:**

The standard treatment of choice for malignant pleural mesothelioma is chemotherapy with pemetrexed and platinum, but the clinical outcome is poor. This study investigates the response to pemetrexed in a panel of eight mesothelioma cell lines and the clinical outcome for patients treated with pemetrexed in relation to folate receptor alpha (FR*α*).

**Methods:**

Cell lines were treated with pemetrexed to determine the concentration that reduced growth to 50% (GI_50_). FR*α* expression was determined by western blotting and that of FR*α*, reduced folate carrier (RFC) and proton-coupled folate transporter (PCFT) by real-time quantitative RT–PCR. Immunohistochemistry for FR*α* was carried out on 62 paraffin-embedded samples of mesothelioma from patients who were subsequently treated with pemetrexed.

**Results:**

A wide range of GI_50_ values was obtained for the cell lines, H2452 cells being the most sensitive (GI_50_ 22 nM) and RS5 cells having a GI_50_ value greater than 10 *μ*M. No FR*α* protein was detected in any cell line, and there was no relationship between sensitivity and expression of folate transporters. FR*α* was detected in 39% of tumour samples, generally in a small percentage of cells. There was no correlation between the presence of FR*α* and the outcome of pemetrexed treatment, and no significant difference between histological subtypes.

**Conclusion:**

Response to treatment with pemetrexed does not depend on the presence of FR*α*.

Malignant pleural mesothelioma (MPM) is an aggressive tumour of the pleura and usually has a poor prognosis. It is increasing in many countries worldwide as a result of widespread exposure to asbestos in the past and is expected to peak around 2015 in the United Kingdom (Hodgson *et al*, 2005). The disease affects more men than women in a ratio of 5 : 1 and tends to have a long latency period of 20–40 years after asbestos exposure, affecting people in the fifth to seventh decade of life. Approximately 80% of MPM is attributed to exposure to asbestos fibres, although some tumours are ‘spontaneous’ with no evidence of asbestos exposure. The outcome of patients with MPM is generally poor, with a median survival of 6–12 months. Most patients are unsuitable for radical surgery. Radiation therapy has been shown to alleviate pain in the majority of treated patients, but the duration of symptom control is short lived, hence chemotherapy is generally the treatment of choice. Clinical trials have shown a partial response rate of 32% using a combination of pemetrexed and carboplatin (Hughes *et al*, 2002). A phase III trial using pemetrexed with cisplatin significantly improved response rates, time to progression, overall survival and quality of life, compared with single agent cisplatin, and suggested that this be used as front-line chemotherapy in MPM patients (Vogelzang *et al*, 2003). Response rate in this study was 41%, with a median survival benefit of 2.8 months, but relapse rates remain high and long-term survival is poor; therefore, improved patient selection for treatment in this disease is still required.

Pemetrexed is a multitargeted antifolate that inhibits several enzymes important in folate metabolism, the main action being on thymidylate synthetase (TS). Inhibition of TS leads to depleted levels of thymidine, which are crucial to DNA synthesis. Pemetrexed also inhibits, to a lesser degree, glycinamide ribonucleotide formyltransferase (GARFT) (Shih *et al*, 1997). Once in the cell, pemetrexed undergoes polyglutamation, often resulting in a tri- or penta-glutamate tail being added to the parent drug. This process is facilitated by the enzyme folyl-poly glutamate synthetase (FPGS), for which pemetrexed has a high affinity, and results in cellular drug retention. The affinity of pemetrexed to TS is also increased by at least 60 times in the polyglutamated form, compared with the parent drug (Hanauske *et al*, 2001).

Folic acid and antifolates enter cells by three mechanisms. The reduced folate carrier (RFC) is a bidirectional anion exchanger that has a high affinity for reduced folate co-factors and antifolates, but a low affinity for folic acid. It has a neutral pH optimum and is ubiquitously expressed (Whetstine *et al*, 2002). The folate receptor alpha (FR*α*) is an energy-dependent high-affinity, low-capacity folate-binding protein that delivers folates and antifolates into cells. The low capacity is due to endocytosis of the receptor that has to return to the cell surface in order to function. Expression is limited to specific tissues and is expressed in a characteristic distribution, for example, on syncytiotrophoblastic cells of the placenta and the proximal convoluted tubule of the kidney (Weitman *et al*, 1992; Mantovani *et al*, 1994). However, FR*α* has been reported to be highly expressed in some malignancies: in 90% of non-mucinous epithelial ovarian cancers (Toffoli *et al*, 1997) and in 72% of malignant mesotheliomas (Bueno *et al*, 2001). Folate receptor alpha has been believed to be important for the physiological transport of folic acid, but not generally for antifolates (Kamen and Smith, 2004). However, there is evidence to suggest that FR*α* may have an important role in folate and antifolate transport under certain circumstances. Studies performed on vulval epithelial cell lines A431 and A431-FBP, a highly expressing FR*α* transfectant, showed that A431-FBP was approximately three-fold more sensitive to pemetrexed than the isogenic non-FR*α-*expressing cell line when cells were maintained in a physiological level of folate (20 nM leucovorin) and was 14-fold more sensitive when maintained in sub-physiological levels (1 nM leucovorin) (Theti and Jackman, 2004). Membrane transport of folates has also been recently reported to occur through a ubiquitously expressed proton-coupled folate transporter (PCFT) (Wang *et al*, 2003; Zhao *et al*, 2008), a low-pH transporter with a high affinity for pemetrexed.

The contribution of FR*α* to pemetrexed transport is likely to be significant in cells in which FR*α* is highly expressed. In these situations, transport of pemetrexed via FR*α* may enhance delivery of the drug to the tumour and potentially enhance response. Recently, a new monoclonal FR*α* antibody has been developed that can be used for immunohistochemistry on formalin-fixed paraffin-embedded (FFPE) tissues (Smith *et al*, 2007) and therefore be more widely used on stored and more readily transportable tumour specimens. This study investigates the growth inhibitory effects of pemetrexed in eight human mesothelioma cell lines and their FR*α* status. It also reports the response to treatment with pemetrexed in mesothelioma patients in relation to FR*α* immunohistochemistry of the tumours.

## Materials and methods

### Cell culture

Eight human mesothelioma cell lines were used in this study. Five cell lines were of epithelioid type: NCI-H28 (H28), NCI-H2052 (2052), NCI-H2452 (2452) (ATCC, Manassas, VA, USA), NCI-H226 (H226) (Cancer Research, London, UK) and JL1 (DSMZ, Braunschweig, Germany); MSTO-211H (MSTO) (ATCC) was of biphasic origin; two cell lines, DM3 and RS5 (DSMZ) were of sarcomatoid type. Cells were grown in RPMI 1640 (R8758, Sigma–Aldrich, Poole, UK) containing 2 mM glutamine, 1.5 g l^−1^ sodium bicarbonate, 4.5 g l^−1^ glucose, 10 mM HEPES, 1 mM sodium pyruvate and either 10% foetal bovine serum (FBS) (Sigma-Aldrich) or 10% dialysed FBS (DFBS) (Invitrogen, Faisley, UK). Dialysis of serum removes low molecular weight compounds such as thymidine and homocysteine, which may affect the response of cells to pemetrexed. The concentration of folic acid in the medium used is 2 *μ*M. All cell lines were subcultured regularly and were negative for mycoplasma.

Growth-inhibition studies using pemetrexed were carried out using the SulfurRhodamine B (SRB) (Skehan *et al*, 1990) assay in 96-well tissue culture plates as previously described (Nutt *et al*, 2004). Cells were seeded at 5 × 10^3^ cells per well and treated with 10 nM to 10 *μ*M pemetrexed for approximately three cell doubling times, apart from the slower growing RS5 and DM3 cells that were treated for 6 days. The concentration of pemetrexed resulting in 50% growth inhibition (GI_50_) for each cell line was calculated using GraphPad Prism software.

Cell lysates required for western blotting were prepared after washing cells with ice-cold PBS and addition of lysis buffer (62.5 mM Tris/HCl pH 6.8, 2% sodium dodecyl sulphate and 10% glycerol).

### Western blotting

Western blotting was carried out to determine the presence and level of expression of FR*α* protein. Cell lysates were prepared and 20 *μ*g aliquots of protein (calculated from quantification using the Pierce BCA protein assay) from each sample were loaded on a 4–20% gradient polyacrylamide gel (Invitrogen). After SDS–PAGE and electroblotting (Towbin *et al*, 1979), membranes were treated for 1 h with TBS-Tween buffer containing 5% milk to block non-specific binding and then incubated with a previously described and validated monoclonal mouse FR*α* primary antibody (Smith *et al*, 2007) for 1 h at room temperature. Secondary HRP-conjugated antibody (goat anti-mouse, Dako, Ely, UK) was used to detect the primary antibodies and antibody-labelled protein bands were visualised by enhanced chemiluminescent detection (ECL) (GE Healthcare, Little Chalfont, UK). Equal loading of protein was verified using a mouse anti-tubulin antibody (Sigma–Aldrich). IGROV1 (an ovarian cancer cell line) cell lysate was used as a positive control and Jurkat cell lysate was used as a negative control for FR*α*.

### Quantitative RT–PCR

RNA was extracted from cells using the Qiagen RNAeasy minikit (Qiagen, Hilden, Germany) and reverse transcribed using TaqMan reverse transcriptase reagents (Applied Biosystems, Warrington, UK). A volume of 2.5 *μ*l cDNA was used for quantitative real-time PCR in a total volume of 10 *μ*l, repeated in triplicate, using the TaqMan gene expression assay Hs00357143_g1 for FR*α*, Hs00953344_m1 for RFC, Hs00611082_m1 for PCFT and Hs99999901_s1 for 18S (all from Applied Biosystems), as well as universal mastermix. The expected amplicon sizes were 89, 106, 72 and 187, respectively. PCR was performed as follows: 50°C for 2 min, 95°C for 10 min, and 40 cycles of 95°C for 15 s and 60°C for 1 min using the ABI Prism 7900HT sequence detection system (Applied Biosystems). Results were quantified relative to 18S, and calibrated to the IGROV1 FR*α* estimation.

### Immunohistochemistry and clinical correlation

Eligible patients were identified from chemotherapy prescription records. All patients underwent histological diagnosis of malignant pleural mesothelioma and were treated according to our institutional protocol, in which carboplatin was administered to produce a value of the area under the curve (AUC) of 5, i.v. over 30 min on day 1 (or cisplatin 75 mg m^−2^, i.v. administered over 3 h on day 1) and pemetrexed administered at a dose of 500 mg m^−2^ i.v. for 10 min on day 1. Cycles were repeated every 21 days to a maximum of six cycles. Patients also received folic acid supplementation at a dose of 400 *μ*g daily, beginning at least 7 days before the first dose of pemetrexed and one injection of 1000 *μ*g vitamin B12 intramuscularly 7–14 days before beginning the treatment. Folic acid was continued, and vitamin B_12_ was repeated every 12 weeks, throughout the treatment until 21 days after the last dose of pemetrexed. Dexamethasone, 4 mg twice daily, was given orally on the day before, the day of and the day after therapy. Our antiemetic regimen included a serotonin antagonist. Chemotherapy was discontinued in the event of disease progression, unacceptable toxicity or after completion of six cycles. Cycles were repeated at 100% of the intended doses if neutrophil and platelet counts were ⩾1.5 × 10^9^ l^−1^ and ⩾100 × 10^9^ l^−1^, respectively. If any dose reduction occurred, patients continued to receive the reduced dose throughout their treatment. All patients underwent planned radiographic evaluation.

Sections of 4 *μ*m were cut from FFPE tissue blocks of identified patients and mounted on SuperFrost Plus microscope slides (VWR International, Lutterworth, UK) and allowed to dry at 56°C. The slides were dewaxed in xylene, rehydrated in graded ethanol and incubated for 10 min in 0.5% hydrogen peroxide solution to block endogenous peroxidase activity. The slides were then treated with an endogenous biotin blocking kit (Dako) before high-temperature antigen retrieval (microwaved thrice for 5 min in citrate buffer pH 6.0). Blocking with a 1 in 5 dilution of normal goat serum for 10 min preceded overnight incubation in a 1 in 20 dilution of FR*α* antibody (Smith *et al*, 2007) at 4°C. Detection of the primary antibody was carried out using biotinylated goat anti-mouse secondary antibody and was visualised using DAB. Between each incubation step, slides were rinsed in TBS pH 7.6 for 2 × 5 min washes. Negative controls with no primary antibody were used and a FFPE section of renal tissue was used as a positive control.

Staining was graded independently by two assessors (JN and AR) using a composite index. The intensity of staining was scored 0–3, with 0 being no staining, 1 weak staining, 2 moderate staining and 3 intense staining. This was multiplied by the percentage of tumour area stained, using 10 for upto 10%, 25 for 11–25%, 50 for 26–50%, 75 for 51–75% and 100 for 76–100% of the tumour area stained, giving a composite score range of 0–300. The staining obtained in the renal tissue was used as a reference for intense staining, with a score of 300.

The composite score for the FR*α* immunohistochemistry of tumour samples was correlated to clinical parameters such as response (objective decrease in tumour size on CT imaging), disease control rate (DCR, presence of stable disease or better on CT), time-to-treatment failure (TTF, time from treatment initiation to documented clinical or radiological progression) and overall survival (OS, time from treatment initiation to death from any cause).

This study was approved by the local research ethics committee and the Newcastle Hospitals Caldicott Guardian.

## Results

### Cell culture

**Figure 1:**
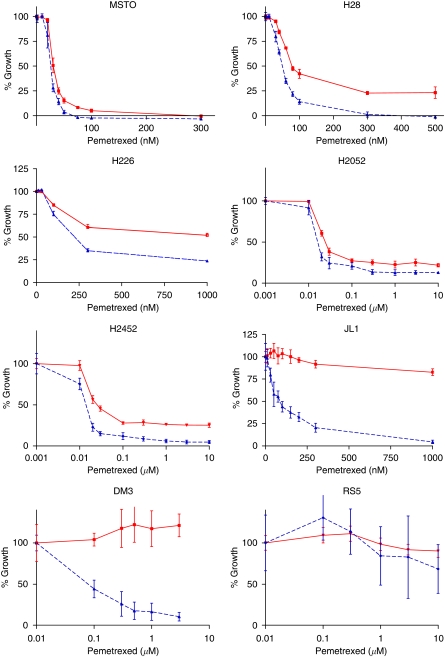
Effect of pemetrexed on growth of mesothelioma cell lines grown in medium containing foetal bovine serum (FBS) (solid line) or dialysed foetal bovine serum (DFBS) (dashed line) with s.d. (*n*=6) for one representative experiment.

Pemetrexed inhibited the growth of mesothelioma cell lines to variable extents. Representative graphs showing patterns of growth inhibition for each cell line in medium containing FBS or DFBS are shown in Figure 1. The mean GI_50_±s.d. value from three replicate experiments is shown in Table 1. GI_50_ values ranged from 14 nM in the H2452 epithelioid cell line to greater than 10 *μ*M in the sarcomatoid cell line RS5. In most cases, apart from sarcomatoid RS5 cells, those grown in medium containing DFBS were more sensitive to pemetrexed than cells grown in medium containing normal FBS, with the largest difference observed in JL1 and DM3 cell lines. This is probably because of the removal of molecules such as folates, thymidine and homocysteine from the serum on dialysis, which are able to rescue cells from the effects of pemetrexed.

### Western blotting and quantitative RT–PCR

**Figure 2:**
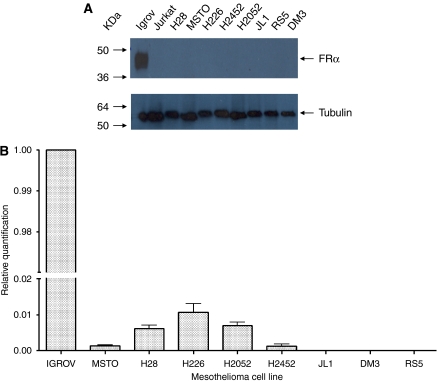
(**A**) Western blot analysis of folate receptor alpha (FR*α*) in mesothelioma cell lysates. IGROV1 was used as a positive control and Jurkat as a negative control. Tubulin antibody shows equal loading in all lanes. Each antibody showed a single band, as shown in the cropped gels. (**B**) Quantification of FR*α* mRNA by real-time PCR in mesothelioma cell lines relative to FR*α-*positive IGROV1 cells.

The results for western blotting and quantitative RT–PCR are shown in Figure 2. In western blotting, there was no evidence of any FR*α* protein in any of the mesothelioma cell lines, but was shown to be present at high levels in the IGROV1 ovarian cell line positive control. Similarly, the results from real-time PCR showed FR*α* mRNA to be undetectable in three cell lines (JL1, DM3 and RS5), whereas extremely low levels at the limit of detection were present in the other mesothelioma cell lines. In these cell lines, the level of expression for FR*α* was approximately 100 times less than the highly expressing IGROV1 ovarian cancer cell line.

**Figure 3:**
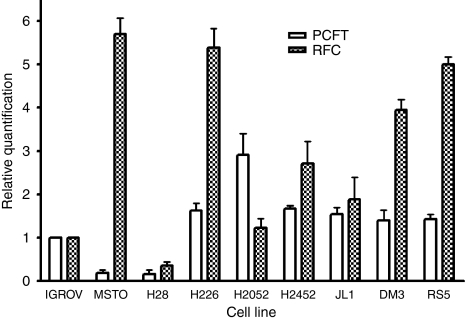
Quantification of reduced folate carrier (RFC) and proton-coupled folate transporter (PCFT) mRNA by real-time PCR in mesothelioma cell lines relative to IGROV1 cells. Error bars show s.d., *n*=3.

The results from real-time RT–PCR for other folate transporters, RFC and PCFT, in the cell lines are shown in Figure 3. The relative expression of both these transporters varied between the cell lines, with the most variation observed in RFC. There was no correlation between RFC and PCFT with the GI_50_ of pemetrexed in the cell lines.

### Immunohistochemistry and clinical correlation

In all, 62 patients were eligible for this study. A summary of demographics and treatment details is shown in Table 2.

**Figure 4:**
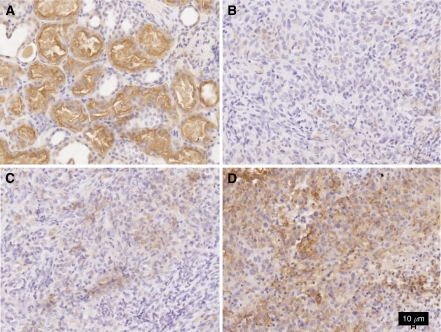
Folate receptor alpha (FR*α*) immunohistochemistry staining of samples of (**A**) renal tissue used as positive control; (**B**–**D**) mesothelioma samples showing weak (**B**), moderate (**C**) and strong (**D**) staining.

A sample of the positive control and various staining intensities are depicted in Figure 4. FR*α* expression was considered to be positive when the composite score was >0. FR*α* expression was positive in 24 of the 62 samples examined (39%, composite score range: 5–142.5). Both membranous and cytoplasmic staining was seen. When present, however, FR*α* expression was usually weak as 12 (50%) stained samples had a score of 30 or less. Only two samples had a composite score of more than 100. FR*α* expression was seen to be higher in the epithelioid disease subtype but was not significant (epithelioid, *n*=16 (44%), biphasic, *n*=6 (35%) and sarcomatoid, *n*=2 (22%), × ^*2*^ 1.614, *P*=0.45).

**Figure 5:**
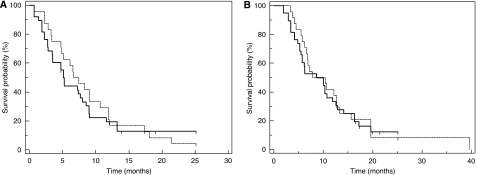
(**A**) Kaplan–Meier time-to-treatment failure (TTF) curves. (**B**) Kaplan–Meier overall survival (OS) curves. Solid line indicates samples with negative folate receptor alpha (FR*α*) expression (*n*=38), dashed line indicates positive FR*α* expression (*n*=24).

There were no significant differences in the proportion of patients with a response or disease control when subdivided into those with a positive or negative expression of FR*α*. Responses were seen in 8 (33%) and 9 (24%) patients with positive and negative FR*α-*stained tumours (*P*=0.56, Fisher’s exact test), whereas 75 and 61%, respectively, had disease control (*P*=0.28, Fisher's exact test). The TTF and OS rates were not statistically different between patients with positive FR*α* tumours and those that were negative (median TTF 7.4 *vs* 5.1 and OS 10.4 *vs* 8.6 months, log rank *P*=0.61 and 0.74, respectively), as shown in Figure 5. The results in terms of treatment response, DCRs, TTF and OS were similar when comparing higher expression (composite score >30) against low or no expression of FR*α*.

## Discussion

Antifolates have been developed over the years to inhibit a number of folate-dependent enzymes. Methotrexate primarily targets dihydrofolate reductase, raltitrexed targets thymidylate synthase and the more recently introduced pemetrexed has multiple targets in the folate pathway. The role of FR*α* in the uptake of both folates (essential for cellular purine and pyrimidine biosynthesis and eventually DNA synthesis) and antifolates has been the subject of numerous publications with contrasting results (Chattopadhyay *et al*, 2004; Theti and Jackman, 2004). Chemotherapy for mesothelioma, using pemetrexed and platinum, is now the standard treatment of choice. In mesothelioma, FR*α* has been described as highly activated (Bueno *et al*, 2001), with 72% of 61 mesotheliomas showing a two- to four-fold higher mRNA expression compared with normal control tissues. Immunohistochemical studies using Mov 18 and Mov 19 antibodies on frozen sections confirmed the results by demonstrating FR*α* at the cellular membrane in 13 of 17 samples. However, a later study (Khokhar *et al*, 2002) demonstrated that despite some expression of FR*α* in human mesothelioma cells, internalisation of methotrexate was predominantly carrier mediated. A highly variable expression of both RFC and FR*α* was found by RT–PCR in a number of mesothelioma samples. With the development of a novel FR*α* antibody for use in paraffin-embedded tissues, in this paper, the role of FR*α* in stored mesothelioma samples has been related to the response of treatment to pemetrexed. The effect of pemetrexed in relation to FR*α* in a selection of mesothelioma cell lines has also been established.

The panel of mesothelioma cell lines used included cells of both epithelioid and sarcomatoid types. Generally, tumours of the sarcomatoid type are less responsive to chemotherapy. This was reflected in the sensitivity of cell lines to continuous pemetrexed treatment, which showed that sarcomatoid cell lines (RS5 and DM3) were less sensitive to pemetrexed than the other cell lines. Clinically, sarcomatoid tumours tend to be more aggressive than epithelioid tumours (Herndon *et al*, 1998). In RPMI medium, sarcomatoid cells were also slower growing (with doubling times of 3–4 days) than epithelioid cells (with doubling times of 1–2 days). A recent report on cell proliferation rates in samples of mesothelioma, using the Ki-67 proliferation index (PI), showed that biphasic tumours had a higher PI than epithelioid and sarcomatoid types, and that the median value for epithelioid samples was higher than that of sarcomatoid tumours (Cakir *et al*, 2006), although only 4.5% of tumours were of the sarcomatoid type.

In all cell lines in which a GI_50_ value was reached, this value was lower when DFBS, rather than normal FBS, was used in the medium. This will have been due to removal of small molecules such as folates, thymidine and homocysteine from the serum on dialysis. A report using pemetrexed and gemcitabine in the NCI-MSTO 211H cell line showed a two-fold higher pemetrexed GI_50_ value of 67 nM (Nagai *et al*, 2008), compared with the value reported here, and strong synergism was observed in these cells when pemetrexed preceded dosing with gemcitabine. Other studies found that the sensitivity of mesothelioma cell lines after 8 h exposure to 30 *μ*M pemetrexed was in the order H2052>H28>MSTO-211H, and that caffeine enhanced pemetrexed activity in the mesothelioma cell lines tested (Min *et al*, 2008). Similar differences in pemetrexed sensitivity have also been reported (Chattopadhyay *et al*, 2006) between H28 and H2052 cells (GI_50_ values 90 and 40 nM, respectively). It has been reported that the effect of pemetrexed is enhanced in cell lines when there is a low or physiological concentration of folate in the medium (Theti and Jackman, 2004) and that higher extra-cellular folate levels correlate inversely with pemetrexed activity *in vitro* in mesothelioma and other solid tumour cell lines (Chattopadhyay *et al*, 2007). In our studies, it was found that when using normal or low-folate medium (results using low-folate medium are not shown) and dialysed or normal serum, the main difference in the effect of pemetrexed was seen between the dialysed serum and normal serum as reported here; hence, low-folate medium was not routinely used.

The RFC, rather than PCFT, shows the most variation in cell lines. There was no correlation between RFC or PCFT with the GI_50_ for pemetrexed. However, a higher RFC was seen in less sensitive cells, apart from the sensitive MSTO cells.

The ratio of epithelioid to sarcomatoid cell lines used in this study reflects the general occurrence of these different tumour types, the majority being epithelioid, 10–15% sarcomatoid and 20–35% biphasic. All cell lines used retained their typical epithelioid or sarcomatoid morphology, using FBS in the medium. Cells are generally grown in medium containing bovine serum, but no changes in morphology were seen, unlike the report of mesothelioma cell lines of different morphology (Klominek *et al*, 1989) established from the pleural fluid of one patient.

Folate receptor alpha was unable to be detected in western blots in any mesothelioma cell line, and was also only barely detectable by real-time PCR. It is therefore evident that the difference in sensitivity to pemetrexed in cell lines is not due to the presence of FR*α* and the uptake of pemetrexed into the cells through FR*α*.

A review on FR*α* (Kelemen, 2006) suggests that further research into the presence of FR*α* in tumours is necessary, as there is a lack of clinical studies using tumour samples, particularly in the context of pemetrexed trials. Until recently, there has been no antibody available to detect FR*α* by immunohistochemistry in paraffin-embedded tumour samples, although the antibodies Mov18 and Mov19 could be used on frozen sections (Bueno *et al*, 2001). Another study of FR*α* in samples of mesothelioma (Khokhar *et al*, 2002) showed no difference in FR*α* gene expression (by RT-RNA) in mesothelioma of differing histological type. Following the development of an FR*α* antibody for use on paraffin-embedded tissues (Smith *et al*, 2007), samples of mesothelioma have been used in this study for FR*α* immunohistochemistry and the results have been compared with response to treatment with pemetrexed. The protein was only detected in 39% of tumours, which is lower than in the previous study, and staining was both membranous and cytoplasmic. Focal staining was generally observed in small regions of tumours, involving only a small percentage of tumour cells and only in two cases was the staining intense. There was no significant difference in FR*α* staining between the three histological subtypes of mesothelioma, and no difference was observed in the overall survival or time to treatment failure for patients with positive FR*α* tumours compared with those with negative FR*α* tumours. FR*α* is therefore unlikely to have a major role in the uptake of pemetrexed in mesothelioma and what little expression there is does not seem to be a predictive marker for treatment response. The low levels of FR*α* expression in malignant pleural mesothelioma samples and the lack of relationship to pemetrexed treatment in this series are consistent with the cell culture observations.

The large differences in response to pemetrexed warrant further investigation. A recent report now provides evidence that the secondary target for the effect of pemetrexed is aminoimidazolecarboxamide ribonucleotide formyl-transferase (AICART), involved in *de novo* purine synthesis, rather than GARFT, (Racanelli *et al*, 2009), and this may explain the unusual activity of pemetrexed in lung cancers.

In conclusion, the use of a varied panel of eight mesothelioma cell lines and the investigation of a large number of tumour samples from patients with mesothelioma treated with pemetrexed in this study demonstrate that there is no relationship between response to pemetrexed and FR*α* expression in mesothelioma, or to the expression of RFC and PCFT in the cell lines. Other reasons for differences in sensitivity to pemetrexed remain to be established, but this study shows that there is no simple relationship between the sensitivity of cell lines to pemetrexed and the expression of any of the folate transporters; other potential molecular markers for pemetrexed response in mesothelioma are currently under investigation.

## Figures and Tables

**Table 1:** Mean GI_50_ values (nM)±s.d. (*n*=3) for pemetrexed in mesothelioma cell lines grown in medium containing foetal bovine serum (FBS) or dialysed FBS (DFBS)

**Cell line**	**MSTO**	**H28**	**H226**	**D2052**	**D2452**	**JL1**	**DM3**	**RS5**
GI_50_ in FBS medium (nM)	33.6±6.2	82.4±3.2	>1000	24±2	22±3	>1000	>10 000	>10 000
GI_50_ in DFBS medium (nM)	27.1±6.4	46.5±3.1	257.3±38	16±1	14±1	102±27	205±96	>10 000

**Table 2:** Patient, disease and treatment demographics

**Parameters**	***n* (%)**
Median age, range	65.6, 49–82
	
*Gender*	
Male/Female	49 (79)/13 (21)
	
*Histology*	
Epithelioid	36 (58)
Biphasic	17 (27)
Sarcomatoid	9 (15)
	
*Stage*	
1/2/3/4	4 (6)/16 (26)/32 (52)/10 (16)
	
*WHO PS*	
0/1/2	8 (13)/36 (58)/18 (29)
	
*Chemotherapy regimen*	
Pemetrexed/Carboplatin	45 (73)
Pemetrexed/Cisplatin	12 (27)
Median chemotherapy cycles (range)	4 (2–6)
